# Vital Signs: Seat Belt Use Among Long-Haul Truck Drivers — United States, 2010

**Published:** 2015-03-06

**Authors:** Guang X. Chen, James W. Collins, W. Karl Sieber, Stephanie G. Pratt, Rosa L. Rodríguez-Acosta, Jennifer E. Lincoln, Jan Birdsey, Edward M. Hitchcock, Cynthia F. Robinson

**Affiliations:** 1 Division of Safety Research, National Institute for Occupational Safety and Health, CDC; 2 Division of Surveillance, Hazard Evaluations, and Field Studies, National Institute for Occupational Safety and Health, CDC; 3 Division of Applied Research and Technology, National Institute for Occupational Safety and Health, CDC

## Abstract

**Background:**

Motor vehicle crashes were the leading cause of occupational fatalities in the United States in 2012, accounting for 25% of deaths. Truck drivers accounted for 46% of these deaths. This study estimates the prevalence of seat belt use and identifies factors associated with nonuse of seat belts among long-haul truck drivers (LHTDs), a group of workers at high risk for fatalities resulting from truck crashes.

**Methods:**

CDC analyzed data from its 2010 national survey of LHTD health and injury. A total of 1,265 drivers completed the survey interview. Logistic regression was used to examine the association between seat belt nonuse and risk factors.

**Results:**

An estimated 86.1% of LHTDs reported often using a seat belt, 7.8% used it sometimes, and 6.0% never. Reporting never using a belt was associated with often driving ≥10 mph (16 kph) over the speed limit (adjusted odds ratio [AOR] = 2.9), working for a company with no written safety program (AOR = 2.8), receiving two or more tickets for moving violations in the preceding 12 months (AOR = 2.2), living in a state without a primary belt law (AOR = 2.1); and being female (AOR = 2.3).

**Conclusions:**

Approximately 14% of LHTDs are at increased risk for injury and death because they do not use a seat belt on every trip. Safety programs and other management interventions, engineering changes, and design changes might increase seat belt use among LHTDs.

**Implications for Public Health:**

Primary state belt laws can help increase belt use among LHTDs. Manufacturers can use recently collected anthropometric data to design better-fitting and more comfortable seat belt systems.

## Introduction

In 2012, motor vehicle crash fatalities (1,153) accounted for 25% of all occupational fatalities (4,628) in the United States.* Of these motor vehicle crash fatalities, 46% of the decedents were truck drivers. In 2012, 2.6 million truck drivers were employed in the United States; 1.7 million drove heavy trucks and tractor-trailers with gross vehicle weight rating (GVWR) >26,000 pounds, and another 840,000 drove medium-sized trucks with GVWR between 10,001 and 26,000 pounds.† The majority of heavy and tractor-trailer truck drivers were long-haul truck drivers (LHTDs), meaning they delivered goods over intercity routes that can span more than one state.

After decreasing to the lowest level ever in 2009, large-truck (GVWR greater than 10,000 pounds)§ occupant deaths have been increasing (1). In 2012, 697 occupants of large trucks died in crashes, and another 26,000 were injured (1). About 41% of truck drivers who lost ≥1 work day from a motor vehicle crash in 2012 missed ≥31 days.¶ Federal regulations require drivers of large trucks to wear a seat belt.** But at least 35% of the truck drivers who died in 2012 were not wearing a seat belt (2). This report estimates the prevalence of seat belt use and identifies factors associated with nonuse of seat belts among LHTDs.

## Methods

In 2010, CDC conducted a nationally representative, personal interview survey of LHTD behavioral characteristics and risk factors at 32 truck stops along interstate highways across the contiguous United States (3). LHTDs were eligible for the survey if they: 1) had driven a truck with three or more axles as their main job for 12 months or more, and 2) took at least one mandatory 10-hour rest period away from home during each delivery run. Eligible drivers were recruited to participate in the survey when they entered the truck stop. Of 3,759 eligible drivers approached, 1,670 (44.4%) participated, of whom 1,265 (75.7%) completed the interview. Self-reported data were collected regarding LHTD crashes, demographics, working conditions, and other risk factors. Details of the sampling design, truck stop selection, survey administration, probability weighting for national estimates, and 95% confidence interval computation have been described (3).

National estimates for LHTD demographics, employment and truck-crash history, and prevalence of seat belt use were analyzed. Logistic regression analyses were performed using unweighted data to examine the association between seat belt nonuse and potential risk factors. Risk factors examined in this analysis included known risk factors (i.e., age, primary enforcement seat belt laws, and sex) and hypothesized risk factors, such as body mass index, ever smoked (yes, no), frequency of driving ≥10 mph over the speed limit (often, sometimes, or never), number of tickets for moving violations received in the preceding 12 months (0, 1, or 2 or more), number of U.S. Department of Transportation recordable crashes†† since working as an LHTD, whether their company had written safety programs (yes, no), and frequency of receiving self-perceived unrealistically tight delivery schedules (often, sometimes, or never) (5,6).

Body mass index was calculated as weight (kg)/height (m)^2^. Values for state primary and secondary laws were derived from drivers’ self-reported state of residence and state data.§§ The logistic regression modeled the probability of never (versus often) using a seatbelt while driving a truck for work. LHTDs who reported sometimes using a seat belt were excluded from the logistic regression analysis because of the binary nature of the outcome variable and because it was not clear whether LHTDs who reported sometimes wearing a seat belt should be combined with LHTDs who reported “often” or “never” wearing a seat belt. LHTDs with missing values for any risk factors of interest also were excluded from analysis. Owner-operators who operated under their own authority were not asked whether their company had a written safety program, and as a result were also excluded from the logistic regression analysis. After all exclusions, data for 1,040 LHTDs were used in the logistic regression analysis.

## Results

Participating LHTDs had a mean age of 47.8 years, and the majority (93.5%) were male (Table 1). Among the drivers, 73.5% were white, 17.1% were black or African American, and 6.9% reported other or multiple races; 8.6% were of Hispanic or Latino ethnicity. An estimated 86.1% of LHTDs often used a seat belt while driving a truck at work, 7.8% used it sometimes, and 6.0% never. On average, these workers had worked 16.4 years as an LHTD. They had worked an average of 60.4 hours in the past 7 days, 46.2 hours of which were spent driving. An estimated 62.9% had slept at home ≤6 days in the past 30 days. On average, they had driven 107,700 miles in the past 12 months. An estimated 34.9% of LHTDs had been involved in at least one crash while working as a LHTD, and 11.9% had been involved in two or more.

Multiple logistic regression results indicated that never using a seat belt while driving a truck was significantly associated with often driving ≥10 mph over the speed limit (adjusted odds ratio [AOR] = 2.9), working for a company that had no written safety policies/programs (AOR = 2.8), receiving two or more moving violation tickets in the preceding 12 months (AOR = 2.2), living in a state without a primary seat belt law (AOR = 2.1), and being female (AOR = 2.3) (Table 2). Never using a seat belt was not significantly associated with body mass index, ever smoking, the number of recordable crashes since working as a LHTD, or perceived unrealistically tight delivery schedules (Table 2).

## Conclusions and Comments

Using a seat belt has been proven to reduce injury and death in the event of a motor vehicle crash for drivers of passenger vehicles and trucks (4,5), and LHTDs are required by federal regulations to use a seat belt.¶¶ Findings from this survey suggest, however, that approximately 14% of LHTDs never or only sometimes use a seat belt. This, coupled with the fact that 34.9% of LHTDs had been involved in at least one U.S. Department of Transportation recordable crash while working as an LHTD and 11.9% had been involved in two or more crashes, underscores the importance of wearing a seat belt.

Never using a seat belt was significantly associated with the absence of a primary enforcement seat belt law in the LHTD’s state of residence. As more states have added primary enforcement seat belt laws, observed seat belt use for drivers of large trucks and buses also has increased (48% in 2003 to 84% in 2013) (5,6). Similar findings have also been reported for belt use by auto drivers by state (7). Belt use among LHTDs might increase further if all states were to adopt primary enforcement belt laws.

Results of this survey also showed a significant association between never using a seat belt and the absence of a written employer safety program. A requirement that drivers and all passengers use their seat belts is an important component of a comprehensive motor vehicle safety management program. Companies can establish and enforce belt-use requirements and give incentives or recognition for compliance or consequences for noncompliance (8). Involving workers in development and implementation of these programs can increase their effectiveness (9,10). In a 2005 survey of truck drivers, 44% reported that their employer imposed no penalties for nonuse of seat belts, and 43% indicated that their employer offered no educational or incentive programs to promote seat belt use (11). Comprehensive safety programs also can address unsafe driving behaviors such as speeding and other moving violations, both of which were found in this survey to be associated with never using a seat belt.

Engineering and design changes also might increase seat belt use among LHTDs. Previous studies identified personal choice and discomfort related to belt positioning, tightness, range of motion, and rubbing as primary reasons not to wear a seat belt (11). It was also reported that seat belts in trucks were uncomfortable for women and shorter drivers (5). CDC recently collected anthropometric data from a nationally representative sample of 1,950 truck drivers (1,779 males and 171 females) (12). These new data can be used by vehicle manufacturers to develop better fitting and more comfortable seat belt systems. Improvements in belt design might help increase belt use among LHTDs, especially female truck drivers, who were shown in this survey to be more likely than males to never use a seat belt.

In addition to nonuse of seat belts, other risk factors, notably drowsy and distracted driving, have been linked to fatal large-truck crashes. A case-control study comparing fatal and nonfatal truck crashes using collision reports for 1998–2002 in Kentucky found that the odds of a fatal crash were 8.2 times higher when the truck driver was unbelted, 3.2 times higher when the truck driver was distracted, and 21 times higher when the truck driver was fatigued or fell asleep (13). In addition to ensuring that truck drivers follow federal regulations*** that limit hours of driving, employers can help reduce drowsy driving by allowing enough time for regular rest. Employers can provide education to increase drivers’ awareness of the impact of long work hours and driving at night on driver fatigue. Free online fatigue management training is available for managers and drivers.†††

The findings in this report are subject to at least six limitations. First, because this was a cross-sectional study, causality could not be determined. Second, the survey was conducted at truck stops, which might be more likely to be used by independent owner-operators and drivers for small companies. Drivers for large companies are more likely to stop at company terminals. Third, self-reported data are subject to recall and interviewer bias. To minimize these biases, this survey employed experienced interviewers, standard interview protocols, and survey-specific training. Fourth, findings might be biased away from the null because respondents might have provided socially and legally appropriate answers to questions regarding speeding, moving violations, or seat belt use. This “social desirability” bias was minimized by the anonymous nature of this survey and by assuring respondents that results would be published only in aggregate form. Fifth, nonresponse bias is possible because only one of three eligible drivers asked to participate completed the interview. Finally, results of the logistic regression analysis might not be applicable to owner-operators who operated under their own authority.

Truck driver safety is important for public health because of the high death toll of truck crashes among both drivers and occupants of other vehicles and the economic burden of truck crashes on society. An estimated 317,000 motor vehicle crashes involving a large truck were reported to police in the United States in 2012 (1). In the aggregate, for each large-truck driver death, six other persons (persons in other vehicles, pedestrians, or cyclists) died in truck crashes (1). Fatal motor vehicle crashes involving large trucks and buses cost the U.S. economy an estimated $40 billion in 2012. The total cost, $99 billion, is much higher when crashes with injuries or property damage are also included (14). Improving truck driver safety calls for multifaceted interventions that include federal regulations, state traffic laws, employer safety programs, improved individual driving behaviors, and updated vehicle designs. To increase seat belt use and reduce drowsy and distracted driving, employers can establish and enforce comprehensive safety programs with belt-use requirements, emphasize belt use in training and safety meetings, schedule adequate rest periods, and prohibit texting or using a handheld phone while driving. States and law enforcement officials can mount targeted and high-visibility enforcement efforts. Vehicle manufacturers can use new anthropometric data to design truck cabs and seat belt systems that better fit contemporary drivers (12).

## Figures and Tables

**TABLE 1:** Selected demographic and employment characteristics, and truck crashes among long-haul truck drivers (LHTDs) — United States, 2010

Characteristic	No. of LHTDs responding	Weighted national estimate*	(95% confidence interval)
**Mean age (yrs)**	1,265	47.8 yrs	(46.4–49.1)
**Mean number of yrs worked as an LHTD**	1,265	16.4 yrs	(14.4–18.5)
**Sex**			
Male	1,184	93.5%	(91.3–95.6)
Female	81	6.5%	(4.4–8.7)
**Hispanic or Latino ethnicity**	106	8.6%	(5.2–12.1)
**Race**			
White	923	73.5%	(69.9–77.2)
Black or African American	196	17.1%	(10.6–23.6)
Other or multiple race†	106	6.9%	(3.4–10.4)
Unknown	40	2.5%	(0.5–4.5)
**Employment**			
Company employee	816	64.5%	(59.7–69.4)
Owner-operator who leased to a motor carrier	360	28.0%	(22.4–33.6)
Owner-operator who operated under own authority	99	7.4%	(3.6–11.3)
**Mean no. of hrs worked in the past 7 days**	1,265	60.4 hrs	(56.3–64.5)
**Average hrs on task in the past 7 days**			
Driving	1,265	46.2 hrs	(44.2–48.2)
Waiting for dispatcher, completing paperwork	968	7.3 hrs	(6.0–8.6)
Loading/unloading/securing the load	592	2.9 hrs	(1.8–3.9)
Truck maintenance	553	1.8 hrs	(0.7–2.8)
**No. of days sleeping at home in past 30 days**			
0	250	18.3%	(14.1–22.5)
1–6	558	44.6%	(39.8–49.5)
≥7	456	37.1%	(30.3–43.9)
**Hrs usually driven before stopping for a break or fuel**			
≤4	594	49.3%	(45.9–52.6)
5–8	545	42.0%	(39.6–44.4)
≥8	109	7.2%	(5.2–9.3)
**Mean miles driven in past 12 months**	1,262	107,700	(101,400–113,900)
**How often do you wear a seat belt while driving a truck at work?**			
Often	1,078	86.1%	(81.6–90.7)
Sometimes	102	7.8%	(6.5–9.1)
Never	82	6.0%	(2.3–9.8)
**Number of DOT recordable truck crashes since working as an LHTD**			
≥2	151	11.9%	(8.1–15.8)
1	285	23.0%	(18.5–27.5)
0	829	65.1%	(61.2–69.0)

**Abbreviation:** DOT = U.S. Department of Transportation.

* Weighted national estimates using 1,265 survey responses.

† Other race includes Asian, American Indian/Alaska Native, Native Hawaiian or Pacific Islander.

**TABLE 2:** Seat belt use among U.S. long-haul truck drivers (LHTDs), by selected characteristics — United States, 2010

Characteristic*	No. of LHTDs used in the regression analysis (n = 1,040)	No. of LHTDs who reported using a seat belt often	No. of LHTDs who reported never using a seat belt	Univariate model		Multivariate model	
	
				COR	(95% CI)	AOR	(95% CI)
**Sex**							
Female	69	61	8	1.9	(0.9–4.1)	2.3†	(1.02–5.3)
Male	971	908	63	Ref		Ref	
**Body mass index**							
Extremely obese	135	128	7	0.6	(0.2–1.6)	0.7	(0.2–1.9)
Obese	465	431	34	0.9	(0.4–1.8)	0.9	(0.4–1.9)
Overweight	320	300	20	0.7	(0.3–1.6)	0.7	(0.3–1.7)
Normal weight	120	110	10	Ref		Ref	
**Ever smoked**							
Yes	712	655	57	2.0†	(1.1–3.6)	1.8	(0.99–3.4)
No	328	314	14	Ref		Ref	
**Number of DOT recordable crashes since working as an LHTD**							
≥2	115	106	9	1.4	(0.6−2.9)	1.4	(0.6−3.1)
1	228	207	21	1.6	(0.9−2.8)	1.7	(0.9−3.0)
0	697	656	41	Ref		Ref	
**Received unrealistically tight delivery schedule**							
Often	189	168	21	2.9†	(1.4–6.3)	2.2	(0.97–5.0)
Sometimes	581	542	39	1.7	(0.9–3.4)	1.7	(0.8–3.4)
Never	270	259	11	Ref		Ref	
**Drive 10 mph or more over the speed limit**							
Often	56	45	11	4.5†	(2.2–9.4)	2.9†	(1.3–6.7)
Sometimes	224	203	21	1.9†	(1.1–3.3)	1.5	(0.8–2.7)
Never	760	721	39	Ref		Ref	
**Number of moving violations received in the past 12 mos**							
≥2	67	56	11	3.0†	(1.5–6.0)	2.2†	(1.04–4.7)
1	133	125	8	1.0	(0.5–2.1)	0.9	(0.4–2.0)
0	840	788	52	Ref		Ref	
**Company has written safety programs**							
No	158	135	23	3.0†	(1.7–5.0)	2.8†	(1.5–5.0)
Yes	882	834	48	Ref		Ref	
**State of residence has primary seat belt use law**							
No	253	227	26	1.9†	(1.1–3.1)	2.1†	(1.2–3.6)
Yes	787	742	45	Ref		Ref	

**Abbreviations:** COR = crude odds ratio; AOR = adjusted odds ratio; CI = confidence interval; Ref = referent; DOT = U.S. Department of Transportation.

* Age was examined as a continuous variable in the model and was not found to be significantly associated with seat belt use (p=0.2).

† COR and AOR are statistically significant at p<0.05 level. COR and AOR are modeling the probability of reporting never using a seat belt.

## Footnotes

* The Bureau of Labor Statistics administers the Census of Fatal Occupational Injuries, which enumerates all fatal occupational injuries in the United States using multiple data sources. The most recent final Census of Occupational Injuries data are for the year 2012 and are posted on the Bureau of Labor Statistics website ( http://www.bls.gov/iif/oshcfoir1.htm ) in several tables, including: Table A-2. Fatal occupational injuries resulting from transportation incidents and homicides, all United States, 2012, and Table A-6. Fatal occupational injuries resulting from transportation incidents and homicides by occupation, all United States, 2012.

† The Bureau of Labor Statistics Occupational Outlook Handbook provides estimated numbers of jobs by occupation. Additional information regarding transportation and material moving occupations is available at http://www.bls.gov/ooh/transportation-and-material-moving/home.htm.

§ Federal statistics on truck crashes combine data for medium (10,001–26,000 pounds gross vehicle weight rating) and heavy (>26,000 pounds) trucks into the single category, large trucks.

¶ The Bureau of Labor Statistics conducts the annual Survey of Occupational Injuries and Illnesses from a nationally representative sample of employer-collected records to estimate occupational injuries and illnesses, including those resulting in >1 day away from work. These data do not include self-employed workers such as independent truck drivers. The BLS provided these unpublished data at CDC’s request.

** 49 CFR § 392.16: Use of seat belts. Washington, DC: Federal Motor Carrier Safety Administration; 1995. Available at http://www.fmcsa.dot.gov/regulations/title49/section/392.16.

†† A U.S. Department of Transportation recordable crash occurs when the crash results in one of the following: a fatality; an injury to a person requiring immediate treatment away from the scene of the accident; or disabling damage to a vehicle, requiring it to be towed.

§§ State primary seat belt laws allow law enforcement officers to issue tickets for nonuse of a seat belt; states with secondary seat belt laws can only issue tickets in conjunction with another traffic offense. At present, 33 states and the District of Columbia have primary seat belt laws for front seat occupants, 16 states have secondary laws, and one state has not enacted a primary or a secondary seat belt law for adults. Additional information available at http://www.ghsa.org/html/stateinfo/laws/seatbelt_laws.html.

¶¶ 49 CFR § 392.16: Use of seat belts. Washington, DC: Federal Motor Carrier Safety Administration; 1995. Available at http://www.fmcsa.dot.gov/regulations/title49/section/392.16.

*** 49 CFR § 395.3: Maximum driving time for property-carrying vehicles. Washington, DC: Federal Motor Carrier Safety Administration; 2013. Available at http://www.fmcsa.dot.gov/regulations/title49/section/395.3.

††† North American Fatigue Management Program. A comprehensive approach for managing commercial driver fatigue; 2015. Available at http://www.nafmp.com/en.
